# Controlled Synthesis of Poly(pentafluorostyrene-ran-methyl methacrylate) Copolymers by Nitroxide Mediated Polymerization and Their Use as Dielectric Layers in Organic Thin-film Transistors

**DOI:** 10.3390/polym12061231

**Published:** 2020-05-29

**Authors:** Alexander J. Peltekoff, Mathieu N. Tousignant, Victoria E. Hiller, Owen A. Melville, Benoît H. Lessard

**Affiliations:** Department of Chemical & Biological Engineering, University of Ottawa, 161 Louis Pasteur, Ottawa, ON K1N 6N5 1, Canada; alexander@peltekoff.com (A.J.P.); mtous011@uottawa.ca (M.N.T.); vhill069@uottawa.ca (V.E.H.); omelv065@uottawa.ca (O.A.M.)

**Keywords:** nitroxide mediated polymerization (NMP), Fluoropolymer, dielectric, organic thin-film transistors (OTFT), interface engineering

## Abstract

A library of statistically random pentafluorostyrene (PFS) and methyl methacrylate (MMA) copolymers with narrow molecular weight distributions was produced, using nitroxide mediated polymerization (NMP) to study the effect of polymer composition on the performance of bottom-gate top-contact organic thin-film transistors, when utilized as the dielectric medium. Contact angle measurements confirmed the ability to tune the surface properties of copolymer thin films through variation of its PFS/MMA composition, while impedance spectroscopy determined the effect of this variation on dielectric properties. Bottom-gate, top-contact copper phthalocyanine (CuPc) based organic thin-film transistors were fabricated using the random copolymers as a dielectric layer. We found that increasing the PFS content led to increased field-effect mobility, until a point after which the CuPc no longer adhered to the polymer dielectric.

## 1. Introduction

Organic thin-film transistors (OTFTs) are expected to be an integral component of next-generation electronic devices due to their beneficial qualities such as reduced manufacturing-cost, lightweight and flexibility. As charge transport in OTFTs is limited to the sub-nanometer interfacial region between the semiconducting and dielectric layer [1,2,3], it is crucial to develop these two materials in parallel to better control their interface, and ultimately engineer higher-performing devices. During fabrication, the surface chemistry of the dielectric layer in bottom-gate configuration will dictate the growth of the semiconductor film, as well as influence the structural packing, domain structures and grain boundaries of the final film [4,5,6]. Hydrophilic surfaces, such as common inorganic oxides like SiO_2_ or Al_2_O_3_, can unfavorably affect the growth mode of organic semiconducting films, as well as providing an abundance of water-absorbing sites that act as a charge carrier traps, resulting in poor device performance [5,7]. Therefore, hydrophobic polymers are frequently used to shield the hydroxyl functional groups on the inorganic oxide dielectric, leading to improved organic semiconductor morphology [1,8,9,10].

Among hydrophobic polymers, those containing fluorinated monomers, such as 2,3,4,5,6-pentafluorostyrene (PFS), have been used to improve the surface characteristics of dielectric layers in OTFTs. Park et al. demonstrated that using poly(PFS) as an interlayer in bottom-gate top-contact pentacene-based transistors improved gate-bias stability [11]. Jang et al. used poly(styrene-*random*-PFS) copolymers to control the dielectric surface energy for high performing OTFTs [12]. The authors reported decreases in field-effect mobility, as the difference in interfacial energy between the dielectric surface and pentacene semiconductor increased. More recently, Jeong et al. used poly(PFS) homopolymer to coat BaTiO_3_ dielectrics, leading to improved OTFT performance and operational stability [13]. They observed a reduction in leakage current, an increase in pentacene grain size and a decrease in hole injection barrier due to favorable surface energetics. While these examples demonstrate poly(PFS)’s ability to tune the inorganic dielectric/organic semiconductor interface, it is desirable to move towards an all-organic solution-processable device for the large scale adoption of OTFT technologies.

When targeting an all-organic dielectric layer, the copolymers must be prepared by a living or controlled free-radical polymerization, to ensure the homogeneity of the final material. Heterogenous copolymers will lead to different and unpredictable self-assembly, ultimately resulting in different material properties [14]. It is critical to be able to predict the polymer properties to better engineer the surface properties of the films formed by the polymers. Fluoropolymers are known to segregate to the surface during elevated temperature processing steps [15,16], further suggesting a homogeneous polymer is required to avoid unwanted phase separation. 

In this study, we develop a dielectric polymer that has tunable surface properties for improved device performance. We report the copolymerization of methyl methacrylate (MMA), a well-known polymer dielectric, with PFS, a unit which will tune the surface chemistry of the dielectric. The controlled copolymerization of PFS [17] has been reported, using reversible addition−fragmentation chain-transfer polymerization (RAFT) copolymerization with styrene [18], by atom transfer radical polymerization (ATRP), to produce block copolymers with MMA [19] and styrene [20], as well as nitroxide mediated polymerization (NMP) in both homopolymerizations [21], and as a comonomer with methacrylic acid (MAA) [22]. We report the reaction engineering of the MMA/PFS system and characterize the resulting polymers. The resulting MMA/PFS copolymers are then integrated into capacitors and transistors, and characterized as a function of copolymer composition. 

## 2. Materials and Methods

### 2.1. Materials

N-(2-methylpropyl)-N-(1-diethylphosphono-2,2-dimethylpropyl)-O-(2-carboxyprop-2-yl) hydroxylamine (MAMA-SG1) (BlocBuilder-MA) was obtained from Prof. Marc Dubé (University of Ottawa), who sourced it from Arkema (Colombes, France). N-tert-butyl-N-(1-diethoxyphosphoryl-2,2-dimethylpropyl)aminooxyl (SG1) was synthesized following a literature procedure from Hlalele et al. [23]. 2,3,4,5,6-Pentafluorostyrene (PFS, 98%) was purchased from Oakwood Chemical (Estil, SC, USA). 2-Butanone (99%), methyl methacrylate (MMA, 99%) and poly(methyl methacrylate) (poly(MMA), *M*_W_ = 120,000 g/mol) were purchased from Sigma-Aldrich (St. Louis, MO, USA). Xylenes (98.5%) were purchased from Anachemia (Quebec, ON, Canada), while hexanes (99%), methanol (99%) and tetrahydrofuran (THF, 99%) were purchased from Caledon Chemical (Caledon, ON, Canada). Prefabricated glass/quartz substrates were purchased from Ossilla (Sheffield, UK). Prefabricated one-inch by one-inch glass substrates were purchased from university wafers. Chromium (Cr, 99.99%) and silver (Ag, 99.99%) electrode metals were sourced from Angstrom Engineering (London, ON, Canada). Copper phthalocyanine (CuPc, 90%) was purchased from TCI Chemicals (Tokyo, Japan) and purified using train sublimation before use.

### 2.2. Synthesis of 2,3,4,5,6-pentafluorostyrene/methyl Methacrylate (PFS/MMA) Random Copolymers

**Figure 1:**
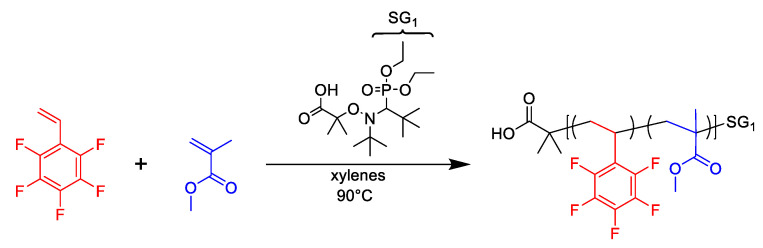
Chemical reaction scheme of 2,3,4,5,6-pentafluorostyrene/methyl methacrylate nitroxide mediated copolymerizations.

The synthesis of PFS/MMA copolymers is shown in Figure 1. The copolymerizations were completed in a three-neck round-bottom flask, equipped with a condensation column and thermocouple, which was used to modulate the temperature of the heating mantle and reactor. A series of experiments of molar compositions of PFS relative to MMA is provided in Table 1. The synthesis of PFS/MMA-20/80 is provided as an example: MAMA-SG1 (0.153 g, 0.40 mmol), SG1 (0.0177 g, 0.06 mmol), xylenes (10 g), PFS (3.26 g, 16.8 mmol), MMA (6.74 g, 67.3 mmol), were added to the reactor with a stir bar. The reaction mixture was stirred and bubbled with nitrogen for 30 min. The nitrogen purge was removed from the mixture while still maintaining an N_2_ atmosphere for the entirety of the reaction. The mantle was heated to 90 °C and t = 0 min was set to when the temperature in the reactor reached 90 °C. The samples were taken periodically by syringe throughout the polymerization. After stopping the reaction and allowing it to cool, the reaction mixture was precipitated into methanol, then re-dissolved into THF and re-precipitated into hexanes, filtered and dried in a vacuum oven at 60 °C overnight to give the final product. The number-average molecular weight ${\bar{M}}_{n}$ = 13.8 kg mol^−1^, dispersity (${\bar{M}}_{w}/{\bar{M}}_{n}$ = 1.15 and PFS molar fraction in the copolymer *F_PFS_* = 0.31).

### 2.3. Metal-Insulator-Metal (MIM) Capacitor Fabrication

Metal-insulator-metal (MIM) capacitors were prepared in an atmospheric environment. First, glass substrates (1” × 1”) were washed by sonicating in a sequence of solvents; soapy water, water, acetone, then methanol, for 5 min each, then dried by blown nitrogen. Silver electrodes (60 nm) were deposited on the prepared glass, using a shadow mask and physical vapor deposition. Then, the solution deposition of the polymer layer was achieved by spin-coating a 50 mg/mL poly(PFS-*ran*-MMA) in methyl ethyl ketone (MEK, 99%) solution at 2000 rpm, followed by an annealing step (150 °C, under vacuum, 1 h). The procedure was then repeated: the same fluoropolymer solution was applied again on the film-coated substrates, and annealing repeated to form the final pin-hole free insulating film of approximately 500 nm. Next, the top Ag electrodes (60 nm) were evaporated, producing the completed MIM capacitors. The perpendicular overlap of the bottom and top Ag electrodes resulted in 10 unique devices of differing areas from 0.35 mm^2^ to 2.88 mm^2^**.**

### 2.4. Bottom-Gate Top-Contact (BGTC) Thin-Film Transistor Fabrication

Bottom-gate top-contact (BGTC) thin-film transistors were fabricated under atmospheric conditions on 20 mm × 15 mm Ossila quartz coated glass substrates. The glass substrates were cleaned by bath sonication: soapy water, distilled water, acetone then methanol, each for 5 min. Similar to the MIM fabrication, a 60 nm Ag gate was patterned on the cleaned substrates by PVD followed by spin-coating of solutions (50 mg/mL in MEK) of poly(PFS-ran-MMA) at 2000 rpm for 1.5 min, followed by thermal vacuum annealing at 150 °C for 1 h. This process was then repeated on the same polymers to give a final polymer layer with a thickness of approximately 500 nm. A 50 nm layer of copper phthalocyanine (CuPc) was then deposited on the PFS/MMA surface by PVD, using a shadow mask. The top Ag source-drain electrodes (60 nm) were then deposited on top of the polymer. This process created 20 individual transistors with a channel length of 1000 μm and a channel width of 30 μm. 

### 2.5. Characterization

**Figure 2:**
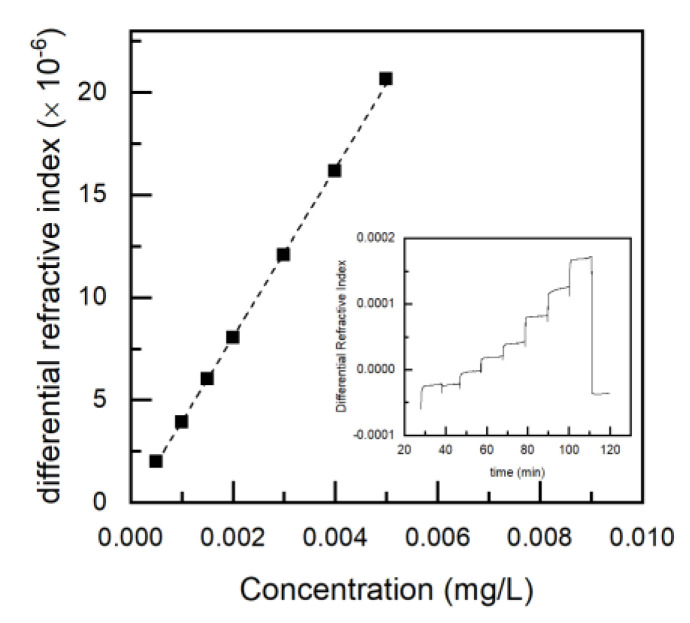
Specific refractive index increment (∂n/∂c) measurement of poly(2,3,4,5,6-pentafluorostyrene) (poly(PFS)). Sample: PFS/MMA-100; *M*_w_ = 19.9 kg mol^−1^; tetrahydrofuran (THF), 30 °C, ($\lambda_{0}$) = 690 nm; 0.5 mL min^−1^; 0.1–5 mg mL^−1^. Inset) Differential refractive index (dRI) of polymer dilution in THF, prior to solvent baseline subtraction of seven solutions of increasing concentrations against time. The initial and final steps represent the THF without polymer dissolved. Main figure dRI post baseline subtraction of poly(PFS) homopolymer solutions plotted against concentration. The slope of the data points (dotted line), determined by a linear fit, corresponds to the ∂n/∂c of PFS in THF, at 30 °C, under 690 nm wavelength). The instrumental standard deviation of each data point issmaller than the size of the data markers.

Polymerization conversion was determined by gravimetry. Polymer compositions were determined by ^1^H NMR and ^19^F NMR spectroscopy, and the use of an α-α-α-trifluorotoluene marker and Bruker AVANCE II 400 MHz spectrometer (Figure S1). Polymer samples were dissolved in chloroform*-d*. In the ^1^H NMR spectra, the integrals of the MMA peak at 3.6 ppm and the marker’s peak at 7.3–7.6 ppm were compared, and in the ^19^F NMR spectra, the integrals of the PFS peak at −144 to −140 ppm and the marker’s peak at −63 ppm were compared. From these two ratios, the composition of poly(PFS-ran-MMA) copolymers was determined. Molecular weight charcteristics of the final copolymers were determined by gel permeation chromatography (GPC)using an Agilent 1260 Infinity at 30 °C, flowing of 1 mL∙min^−1^ of THF, as the eluting solvent via two MZ-Gel SD plus Linear 5 µm, 300 × 8.0 mm^2^ columns. Triple detection was accomplished using a multi-angle light scattering (MALS) detector (DAWN HELEOS II), differential viscometer (ViscoStar II) and a differential index detector (Optilab T-rEX). The specific refractive index increment, ∂n/∂c of the PFS homopolymer was determined via off-line, batch-mode differential refractive index (dRI) experiments. A succession of solutions of sequentially increased concentrations of polymer in THF was injected directly into the dRI detector with a syringe pump, followed by pure THF without any dissolved analyte for a baseline. Figure 2, shows each step change of dRI of a known dilution of increasing poly(PFS) concentrations. The dRIs are measured and plotted (after baseline subtraction from solvent) against the concentration of each concentration (Figure 2 inset), then the data points are fitted linearly to give the ∂n/∂c of the poly(PFS) at 30 °C in THF with a wavelength of 690 nm. The ∂n/∂c values of the poly(PFS) homopolymer and poly(MMA) homopolymer were found to be 0.041 and 0.0831, respectively. 

The ∂n/∂c of copolymers were determined from the ∂n/∂c values of the component homopolymers and the mass fraction of the comonomer in the copolymer. For a generic poly(PFS-ran-MMA) copolymer, its ∂n/∂c is calculated from the equation [24]: ${\left(\frac{\partial n}{\partial c}\right)}_{PFS-MMA}=w_{PFS}{\left(\frac{\partial n}{\partial c}\right)}_{PFS}+w_{MMA}{\left(\frac{\partial n}{\partial c}\right)}_{MMA}$ where $w_{PFS}$ and $w_{MMA}$ are the mass fractions of PFS and MMA in the poly(PFS-ran-MMA) copolymers, and ${\left(\partial n/\partial c\right)}_{PFS}$ and ${\left(\partial n/\partial c\right)}_{MMA}$ are the ∂n/∂c of the pure homopolymers determined under the same experimental conditions. The glass transition temperatures (*T_g_*s) were found using differential scanning calorimetry (DSC; TA Instruments Q2000). A heating/cooling rate of 10 °C/min under a nitrogen atmosphere across a temperature span of 10 °C to 170 °C was performed twice. The *T_g_* was then found by the midpoint from the second thermogram heating cycle. Contact angle measurements were performed on a VCA Optima goniometer (AST Products Inc). Droplets (0.5 µL) of DI water were deposited from a needle, imaged and contact angle were determined using a three-point curve fitting. The thickness of poly(PFS*-co-*MMA) films were measured with a Bruker Dektak XT profilometer. Prepared films were scratched using a diamond tip pen three times, and step edges measured then averaged. Impedance properties of the polymers in a metal-insulator-metal (MIM) structure were measured using electrochemical impedance spectroscopy (EIS; Metrohm PGSTAT204). An equivalent circuit model consisting of a resistor and a capacitor in parallel was used to extract the effective capacitance, which was calculated from the equation: C=1/(2πfIm(Z)), where f is the frequency and Z is the measured impedance. Measurements were conducted over a frequency range of 10^−2^–10^5^ Hz with an AC amplitude of 10 mV under atmospheric conditions. Current-voltage (I-V) characteristics of transistors were measured using a Keithley 2614B, holding the gate-source voltage (*V_GS_*) constant, then sweeping the source-drain voltage (*V_SD_*), while measuring the source-drain current (*I_SD_*). Voltage step increases were set with a delay of 200 ms between measurements. Each device was characterized three times, and the obtained values were averaged. Transfer curves were measured in the saturation regime and were modelled using the following equation: $I_{D}=\frac{W}{2L}C\mu {\left(V_{GS}-V_{th}\right)}^{2}$, where *L* and *W* are the length and width of the channel, respectively; *C* is the capacitance density determined from the thickness and dielectric constant of the gating medium, and *µ* is the field-effect mobility which was calculated from the slope of trendline through the linear region of the square root *I_SD_* plotted against *V_GS_*. All measurements were conducted in the atmosphere at room temperature. 

## 3. Results and Discussion

### 3.1. Copolymer Synthesis: Kinetics & Control

Pentafluorostyrene (PFS) has been successfully homopolymerized [21] using nitroxide mediated polymerization (NMP), as well as other controlled free-radical approaches, such as ATRP and RAFT [18,19,20,25]. Methacrylic monomers are notoriously challenging to polymerize in pseudo-living fashion, due to their large equilibrium constant for propagation and unwanted side reactions [26,27]. To increase the number of living propagating chains methacrylate monomers are often copolymerized with a slowly propagating comonomer, which is more compatible with NMP, such as styrenics [28,29,30,31], acrylonitrile [32,33,34,35] or cyclic ketene acetal 2-methylene-4-phenyl-1,3-dioxolane [36,37]. PFS has been demonstrated to behave as a controlling comonomer in the NMP of various methacrylic monomers, such as methacrylic acid [22], 5-methacryloyloxy-2,6-norboranecarbolactone (NLAM) [38], oligo(ethyleneglycol) methacrylate (OEGMA) [39] and, very recently, methyl methacrylate (MMA) [40]. Delaittre and coworkers demonstrated that ≈5 mol% of PFS could control the copolymerization of MMA, leading to a high MMA-containing pseudo-living macroinitiator, suitable for the chain extension of styrene [40]. To further characterize this copolymerization at low through high PFS loadings we performed a range of copolymerizations with a target number average molecular weight (${\bar{M}}_{n}$) of 25 kg mol^−1^ in a 50 wt% solution of xylenes at 90 °C, with feed compositions ranging from 5–100 mol%, which is detailed in Table 1. The kinetics of NMP is typically characterized by *k*_P_*K* values, where *k*_P_ and *K* represent the propagation rate and activation-deactivation equilibrium constants, respectively. The activation-deactivation constant (*K*) is a function of the propagating radicals, [P•], free nitroxide, [N•] and dormant alkoxyamine-terminated species, [P−N] concentrations. *K* is calculated from Equation (1) below:

(1) $$K=\frac{\left[N•\right]\left[P•\right]}{\left[P-N\right]},\;$$

Numerous assumptions are made to simplify Equation (1) to produce an estimate of the important combination parameter *k*_P_*K* from the kinetic data. Within the initial stages of polymerization, the concentration of free nitroxide radicals is high and relatively constant. Therefore, the initial concentration of additional SG1, [SG1]_0_, is substituted for [N•], ([N•] = [SG1]_0_). The initial molar ratio of SG1 and MAMA-SG1 concentrations is represented by *r* = [SG1]_0_/[MAMA-SG1]_0_ = 0.15 for all polymerizations performed (Table 1), indicating the termination by B-hydrogen chain transfer to SG1 rate is low, and the primary mechanism of irreversible termination is from homotermination [27,41]. Initially, in the polymerization, when polymer chains are of a few repeat units, homotermination is low. We can therefore assume the concentration of reversibly deactivated alkoxyamine-terminated species is equal to the initial concentration of alkoxyamine initiator ([P−N] = [MAMA-SG1]_0_). The assumptions made are valid if polymerization exhibits pseudo-living behavior; molecular weight increases linearly with the conversion. Finally, *k_p_K* is calculated and the initial molar concentrations of SG1 and MAMA-SG1 are substituted by *r* to result in Equation (2):

(2) $$k_{p}K=k_{p}\frac{{\left[SG1•\right]}_{0}\left[P•\right]}{{\left[MAMA-SG1\right]}_{0}}=r\cdot k_{p}\left[P•\right],$$

**Figure 3:**
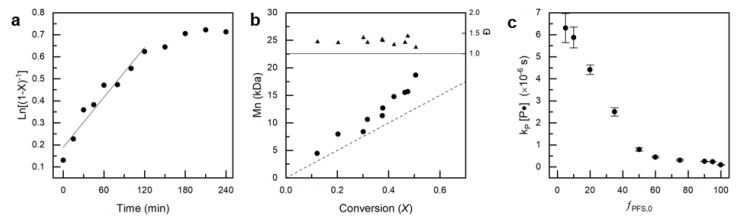
(**a**) Characteristic kinetic plot of $\ln\left[{\left(1-X\right)}^{-1}\right]$ vs copolymerization time for experiment PFS/MMA-05/95. (**b**) Progression of experimental number average molecular weight, $\bar{M_{n}}$, and dispersity, *Đ*, with conversion, *X* for experiment PFS/MMA-05/95. (**c**) Molar feed ratio of PFS, *f_PFS,0_* versus apparent rate constant, *k_p_*[P•] found from the kinetic plots slope.

The slope of the $ln{\left(1-X\right)}^{-1}$ vs time plot is equal to the apparent rate constant, *k_p_*[P•]. In this study, gravimetric analysis was utilized to determine conversion, X. A representative $ln{\left(1-X\right)}^{-1}$ vs time plot used to calculate *k_p_*[P•] from the slope can be found in Figure 3, while all the resulting *k_p_*[P•] values can be found in Table 2. PFS/MMA copolymerizations also demonstrate a linear increase in ${\bar{M}}_{n}$ as a function of *X*, which is consistent with the previous assumptions suggesting Equation (2) is valid (Figure 3). As the PFS content in the feed is increased, the *k_p_*[P•] decreases (Table 2), which is the typical kinetic behavior of MMA/styrenic systems by NMP.

### 3.2. Reactivity Ratio Determination

**Figure 4:**
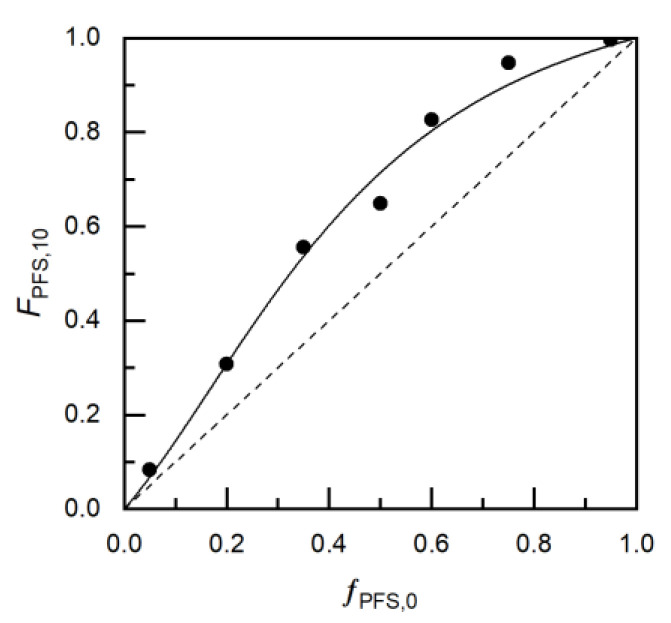
Mayo-Lewis plot of copolymer composition with respect to 2,3,4,5,6-pentafluorostyrene, *F_PFS,10_*, versus monomer feed composition*, f_PFS,0_*. The experimental data for MMA/PFS copolymerization is represented by solid circles, while the solid line shows the fit from the reactivity ratios calculated from the non-linear least-square fitting to the Mayo-Lewis equation (*r*_PFS_ = 3.50 ± 0.70 and *r*_MMA_ = 0.20 ± 0.21). The straight dashed line indicates the azeotropic composition (*f*_PFS,0_ = *F*_PFS_). Table 2 lists the experiments used for the plots.

The final composition of the copolymer will be used to engineer and tune material properties; therefore, it is necessary to have the ability to synthesize a copolymer of a specifically desired composition. Knowledge of the comonomer reactivity ratios is therefore required. To determine the comonomer reactivity ratios, a series of copolymerizations were performed using the same protocol used in kinetic experiments; however, these copolymerizations were purposely ended at low conversion (*X* < 0.10) to avoid compositional drift effects. Seven copolymers with initial PFS/MMA compositions; *f_PFS,0_* = 0.05, 0.20, 0.35, 0.50, 0.60, 0.75 and 0.95 were copolymerized. Copolymer composition was determined by a combination of ^1^H NMR and ^19^F NMR spectroscopy, with the use of an α-α-α-trifluorotoluene marker, and are shown in Figure 4, where the initial molar feeds are displayed against the final copolymer compositions (*X* < 0.10). Reactivity ratios of the comonomers were found with the Mayo-Lewis equation (Equation (3)) [42,43]:

(3) $$F_{PFS}=\frac{r_{PFS}f_{PFS}^{2}+f_{PFS}f_{MMA}}{r_{PFS}f_{PFS}^{2}+2f_{PFS}f_{MMA}+r_{MMA}f_{MMA}^{2}+r_{MMA}f_{MMA}^{2}}\;;\;\;\;\;where,\;\;\;r_{PFS}\;=\frac{k_{p.PFS-PFS}}{k_{p.PFS-MMA}}\;and\;r_{MMA}=\frac{k_{p.MMA-MMA}}{k_{pMMA-PFS}}$$

where the final molar copolymer composition $F_{PFS}$ is a function of initial molar feed compositions *f_PFS,0_* and *f_MMA,0_* and the reactivity ratios $r_{PFS}$ and $r_{MMA}$. *ƒ_PFS,0_* and *F_PFS,10_* tabulated in Table 2, were substituted into Equation (3) for a reactivity ratio determination. A non-linear least squares (NILS) fitting of the Mayo-Lewis equation to the experimentally measured copolymer compositions was performed to converge on the reactivity ratios, which are displayed in Figure 4 [44,45,46]. The fitting shows *r_PFS_* > *r_MMA_*, indicating a preferred addition of the controlling PFS comonomer over MMA suggesting the final copolymers synthesized may have a gradient composition where the initiation end is heavily comprised of the fluorinated sytyrenic, and the propagating end is more MMA rich in comparison. Earlier studies have determined a styrenics capability to maintain a pseudo-living copolymerization of methacrylates is dependent on the reactivity ratios: if the propagation of the styreneic is preferred, a pseudo-living copolymerization can be performed with MMA feeds as significant as *f_MMA,0_* ≈ 0.95–0.99 [47]. However, if the reactivities suggest a preferred propagation of the methacrylate over styreneic, then a pseudo-living copolymerization will only be possible with feeds extremely rich in the controlling styrenic monomer, for instance, methacrylic acid (MAA) and PFS requires *f_PFS,0_* > 0.60 to result in a pseudo-living copolymerization [22]. The results we found indicate that the copolymerization of MMA and PFS will exhibit pseudo-living copolymerization behavior over a wide range of feed compositions.

### 3.3. Characterization of the Final Random Copolymers 

After the polymer reaction engineering was performed, a subset of materials of broad (*F_PFS_* = 0.08–1.00) composition was selected and extensively characterized to determine relationships between the copolymer composition and material property (Table 3). The bulk material properties were characterized by GPC and DSC. The molecular weights of the materials ranged from 19 kDa to 74 kDa, all with a relatively narrow dispersity, as expected by controlled polymerization. The glass transition temperatures, *T_g_*s ranged from (90 to 110 °C, Figure S2).

**Figure 5:**
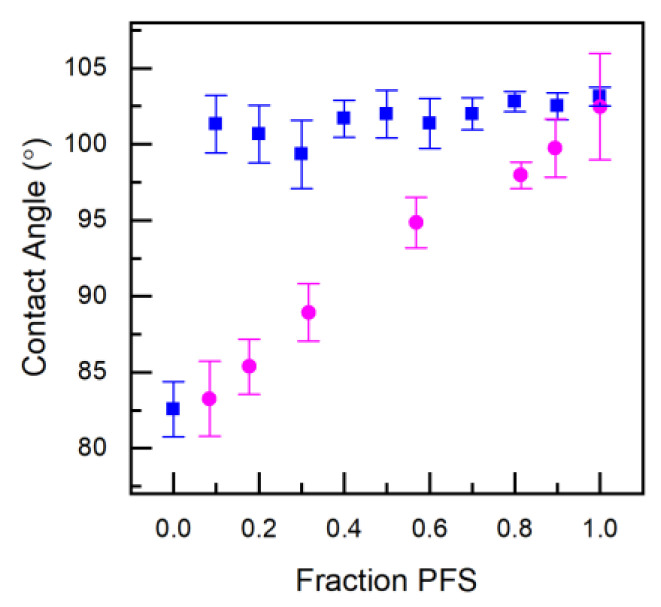
Contact angle measurements of PFS/MMA thin-film copolymers (circles) and PFS/MMA thin-film blends (squares) formed from spin-coating a 50mg/mL solution in MEK solvent after annealing at 150 °C for 2 h.

Contact angle measurements were performed on thin-films of the PFS/MMA copolymers, as well as blends of the respective poly(MMA) and poly(PFS) homopolymers (Figure 5). The blended films possessed a contact angle of around 100°, the same as the PFS homopolymer, which is due to the migration of PFS homopolymer to the surface during annealing steps, this surface segregation of fluorinated components has also been noted in polystyrene and vinyl acetate blended polymer films [15,16]. The contact angles of the copolymer films ranged linearly from 83.3 ± 2.5 to 99.7 ± 1.9 at compositions of *F_PFS_* = 0.08 to 0.89, which falls in between the respective homopolymer chracteristics (Figure 5). These results highlight the necessity of copolymers over simple blending techniques to tune surface properties. 

**Figure 6:**
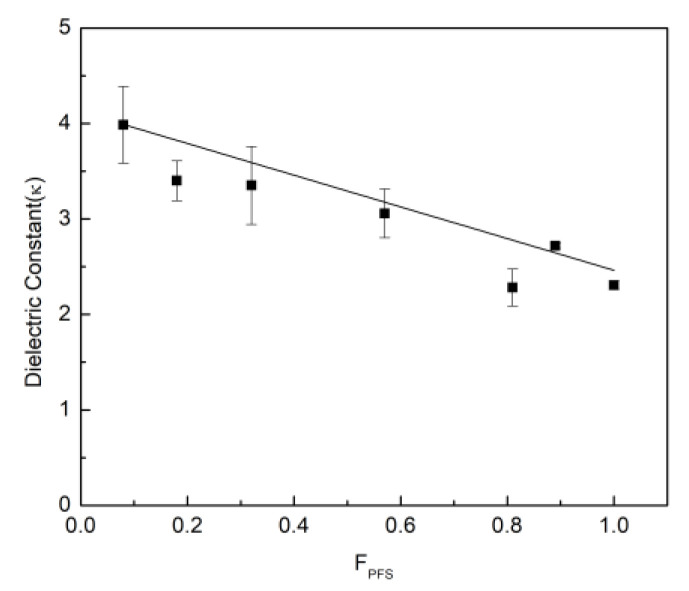
Dielectric constant of copolymer versus copolymer composition, F_PFS._ The solid line is the line of best fit to the experimental data points.

Metal-insulator-metal (MIM) capacitors were fabricated to analyze the dielectric behavior of the copolymers by electrical impedance spectroscopy (EIS). The MIM sandwich structure was fabricated by spin coating the PFS/MMA copolymers between silver electrodes, which were deposited by physical vapor deposition. Using EIS and the film thickness from profilometry, the dielectric constant was determined using the equation:

$$k=\frac{C\cdot d}{\varepsilon_{0}\cdot A}$$

where C is the capacitance [F], d is the insulator thickness [m], A is the area of the electrode area [m^2^] and $\varepsilon_{0}$ is the permittivity constant [F/m]. The dielectric constant was shown to slightly increase at lower frequencies (Figure S3) and exhibited no voltage dependence. As shown in Figure 6, the dielectric constant of the copolymers clearly shows a linear decrease from 3.9 to 2.3, with increasing PFS content at 100 kHz. These results demonstrate that the dielectric properties of the poly(MMA) polymer can be tuned with the copolymerization of a known amount of PFS monomer.

### 3.4. Poly(PFS-ran-MMA) Copolymers as Dielectric Materials in Organic Thin-Film Transistors

**Figure 7:**
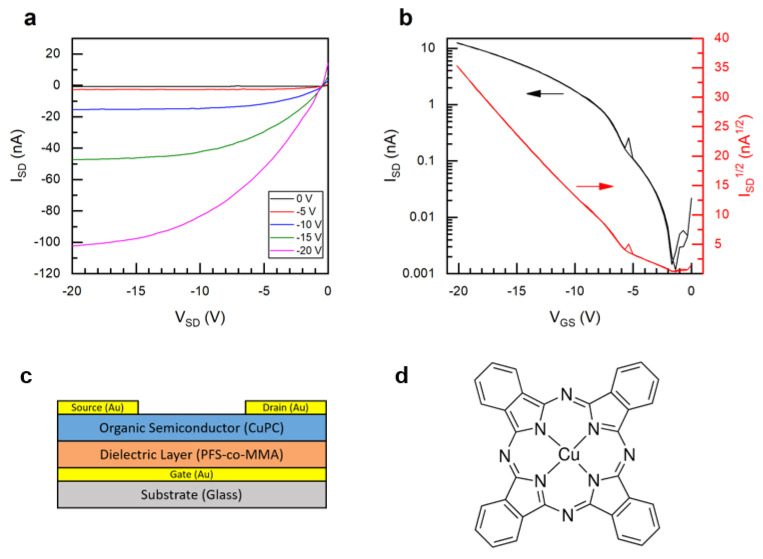
Characteristic (**a**) output and (**b**) transfer curves for (**c**) bottom-gate top-contact (BGTC) organic thin-film transistor (OTFT) devices with (**d**) copper phthalocyanine (CuPc) as the semiconductor (for PFS 57, VSD-20). All data for devices characterized in air.

**Figure 8:**
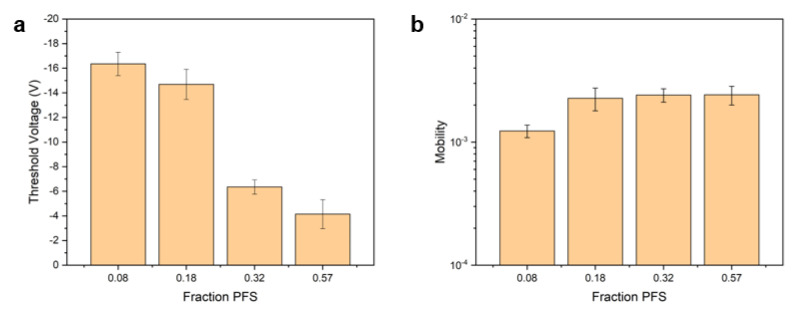
(**a**) Threshold voltage and (**b**) field-effect mobility for CuPc OTFTs with poly(PFS-co-MMA) dielectrics of varying composition.

Bottom-gate top-contact (BGTC, Figure 7) copper phthalocyanine (CuPc) based OTFTs were fabricated using poly(PFS-ran-MMA) copolymers as a dielectric layer to identify the impact of PFS content on the hole transport mobility (*µ*), the on-off current (*I_ON/OFF_*) and the threshold voltage (*V_T_*), and can be found in Table 4. The *µ* of the OTFT devices was calculated using the capacitance densities for the corresponding copolymers obtained from the dielectric constants in the previous section. The resulting OTFTs were characterized by having *μ* = 1 to 3 × 10^−3^cm^2^/Vs, *I_ON/OFF_* 1 to 10 × 10^3^ and a *V_T_* = −4 to −17 V, which is typical for CuPc based OTFT devices [48,49,50]. Characteristic output and transfer curves are shown in Figure 7. Fluorinated surfaces are electron-withdrawing, which have been shown to improve the performance of p-type semiconductors [51]. In this case and highlighted in Figure 8, the increase in the content of PFS reduces the *V_T_*, while having little effect on the *µ*, which is consistent with what we would expect for the addition of fluorinated dielectrics [52]. This suggests that the PFS molecules present on the dielectric surface reduce the concentration of charge trap sites at the semiconductor/dielectric interface [11,12]. For the PFS/MMA copolymers with a PFS composition greater than *F_PFS_* = 0.57_,_ there were few functioning devices, and the results were unreliable with large deviations. We identified that during fabrication, when the polymer was too rich in PFS content, the CuPc semiconductor would no longer form uniform films (Figure S4). This was obvious from the lack of blue color (CuPc, Figures S4 and S5) in the films and the drop in functioning devices (Table 4). The devices that did function when *F_PFS_* > 0.57 were when some CuPc deposited in the channel (Figure S5b). The microstructure of the organic semiconducting layer, especially at the dielectric interface where the conductive channel is formed, has a significant effect on the charge transport properties [53,54,55]. Therefore, we performed powder X-ray diffraction on the CuPc layer, which was deposited on the different copolymers, and the spectra can be found in the ESI (Figure S6). In all cases, we observed a weak characteristic peak corresponding to the CuPc at 2θ = 7°. No significant differences in diffraction intensity (or peak location) between the CuPc films that grew on different surfaces was observed. Further investigation would be necessary to probe the solid-state arrangement of these films and identify the effect of the copolymer composition on the CuPc film growth. Regardless, these results demonstrate that PFS comonomers can be introduced into poly(MMA) dielectrics to tune the surface properties, dielectric properties and the resulting OTFT properties.

## 4. Conclusions

Poly(2,3,4,5,6-pentafluorostyrene) were synthesized in large yields by Nitroxide Mediated Polymerization at 90 °C. The copolymerization exhibited pseudo-living behavior shown by the apparent polymerization rate, which follows first order kinetics with reference to monomer conversion. Additionally, the molecular weight increases in a linear relation to monomer conversion. Finally, the molecular weights experimentally measured align with the theoretical values, and are of relatively low dispersity (*M*_w_/*M*_n_ ≤ 1.3). Thin-film contact angle and dielectric constant were shown to change linearly with copolymer composition. We investigated the effects of copolymer composition with respect to fluorinated content on bottom-gate top-contact CuPc OTFT performance characteristics, and found a reduction in threshold voltage with increasing fluorine content; however, materials which were too rich in fluorine content resulted in poor semiconductor adhesion and non-functional devices.

## Figures and Tables

**Table 1:** Formulations for 2,3,4,5,6-pentafluorostyrene/methyl methacrylate random copolymerizations in 50 wt% xylenes solution at 90 °C initiated by BlocBuilder-MA.

Exp ID ^a)^	MAMA-SG1 ^b)^		SG1 ^c)^		PFS		MMA		Xylenes		
	mmol	mg	mmol	mg	mmol	g	mmol	g	mmol	g	mL
PFS/MMA-05/95	0.4	152.6	0.06	17.7	4.8	0.93	90.6	9.07	94.2	10.0	11.6
PFS/MMA-10/90	0.4	152.6	0.06	17.7	9.1	1.77	82.2	8.23	94.2	10.0	11.6
PFS/MMA-20/80	0.4	152.6	0.06	17.7	16.8	3.26	67.3	6.74	94.2	10.0	11.6
PFS/MMA-35/65	0.4	152.6	0.06	17.7	26.3	5.11	48.9	4.89	94.2	10.0	11.6
PFS/MMA-50/50	0.4	152.6	0.06	17.7	34.0	6.60	34.0	3.40	94.2	10.0	11.6
PFS/MMA-60/40	0.4	152.6	0.06	17.7	38.3	7.44	25.6	2.56	94.2	10.0	11.6
PFS/MMA-75/25	0.4	152.6	0.06	17.7	44.0	8.53	14.7	1.47	94.2	10.0	11.6
PFS/MMA-90/10	0.4	152.6	0.06	17.7	48.7	9.46	5.4	0.54	94.2	10.0	11.6
PFS/MMA-95/05	0.4	152.6	0.06	17.7	50.2	9.74	2.6	0.26	94.2	10.0	11.6
PFS/MMA-100	0.4	152.6	0.06	17.7	51.5	10.00	0.0	0.00	94.2	10.0	11.6

^a)^ Experimental identification (ID) is displayed as PFS/MMA-Y: where PFS and MMA correspond to 2,3,4,5,6-pentafluorostyrene and methyl methacrylate, respectively. The number abbreviation refers to the molar feed of PFS to MMA. ^b)^ N-(2-methylpropyl)-N-(1-diethylphosphono-2,2-dimethylpropyl)-O-(2-carboxyprop-2-yl) hydroxylamine (MAMA-SG1) selected such that target *M*n = 25,000 g/mol. ^c)^ Initial molar concentration ratio of SG1 free nitroxide to MAMA-SG1 = r = [SG1]_0_/[MAMA-SG1]_0_ = 0.15.

**Table 2:** 2,3,4,5,6-pentafluorostyrene/methyl methacrylate (PFS/MMA) random copolymerizations in 50 wt% xylenes solution at 90 °C, using a MAMA-SG1 initiator.

Exp ID ^a)^	*f*_PFS,0_^b)^	*F_PFS,_*_10_^b)^	*k_p_*[P•] (s^−1^) ^c)^	Polymerization Time	Final Conversion ^d)^ (*X*)
PFS/MMA-05/95	0.05	0.08	(6.30 ± 0.66) × 10^−5^	4 h	0.61
PFS/MMA-10/90	0.10	-	(5.88 ± 0.47) × 10^−5^	5 h	0.56
PFS/MMA-20/80	0.20	0.31	(4.41 ± 0.21) × 10^−5^	5 h	0.43
PFS/MMA-35/65	0.35	0.56	(2.50 ± 0.19) × 10^−5^	5 h	0.32
PFS/MMA-50/50	0.50	0.65	(7.93 ± 0.75) × 10^−6^	5 h	0.52
PFS/MMA-60/40	0.60	0.83	(4.47 ± 0.40) × 10^−6^	6 h	0.11
PFS/MMA-75/25	0.75	0.95	(3.07 ± 0.38) × 10^−6^	6 h	0.19
PFS/MMA-90/10	0.90	-	(2.50 ± 0.07) × 10^−6^	7 h	0.08
PFS/MMA-95/05	0.95	0.99	(2.37 ± 0.06) × 10^−6^	7 h	0.08
PFS/MMA-100	100	1.00	(9.03 ± 1.28) × 10^−7^	10 h	0.38

^a)^ Experimental identification (ID) is presented as PFS/MMA-Y: where PFS and MMA correspond to 2,3,4,5,6-pentafluorostyrene (PFS) and methyl methacrylate (MMA), respectively. The number abbreviation refers to the molar feed of PFS to MMA. ^b)^ *f_PFS,0_* is the initial molar fraction of PFS in the feed, and *F_PFS_,10* = copolymer composition at 10% conversion determined by gravimetry. ^c)^ $k_{P}\left[P•\right]$ = apparent rate constant, found using slopes from the linear regions of the kinetic plots. ^d)^ Final conversion determined by gravimetry

**Table 3:** Molecular Weight Distribution Data & Glass Transition for PFS/MMA copolymers.

*F_PFS_* *^1)^*	$\partial n/\partial c{\;}^{2)}$ $\left[mL\;g^{-1}\right]$	${\bar{M}}_{n}\;(\mathrm{Da}){\;}^{3)}$ $\left[g\;mol^{-1}\right]$	${\bar{M}}_{w}\;(\mathrm{Da}){\;}^{3)}$ $\left[g\;mol^{-1}\right]$	Đ ^3)^ $Đ{\bar{M}}_{w}/{\bar{M}}_{n}\rceil$	*T_g_* ^4)^ [°C]
0.08	0.0767	21,100	25,310	1.20	110
0.18	0.0706	14,720	18,970	1.29	107
0.32	0.0632	13,790	15,860	1.15	100
0.57	0.0529	10,650	11,910	1.13	90
0.81	0.0455	10,550	13,060	1.24	88
0.89	0.0435	51,700	73,250	1.42	84
1.00	0.0411	17,400	19,900	1.14	79

*^1)^* Final molar copolymer composition, $F_{PFS}$ ^2)^ ∂n/∂c used was weight average of the homopolymers (0.0411 for poly(PFS) and 0.0831 for poly(MMA)) ^3)^ Number-average molecular weight (${\bar{M}}_{n}$), weight-average molecular weight (${\bar{M}}_{w}$) and the dispersity (Đ = Mw/Mn) were determined by gel permeation chromatography (GPC)*. ^4)^ glass transition temperature determined by differential scanning calorimetry (DSC).

**Table 4:** Bottom-gate top-contact (BGTC) copper phthalocyanine (CuPc) based OTFTs fabricated using poly(PFS-ran-MMA) copolymers as the dielectric layer.

*F_PFS_^a)^*	μ (cm^2^/Vs) ^b)^	I_ON/OFF_^b)^	*V_T_* (V) ^b)^
0.08	1.23 ± 0.144 × 10^−3^	7.51 × 10^3^	−16.35 ± 0.94
0.18	2.27 ± 0.473 × 10^−3^	1.83 × 10^4^	−14.69 ± 1.22
0.32	2.41 ± 0.301 × 10^−3^	3.37 × 10^4^	−6.35 ± 0.58
0.57	2.42 ± 0.423 × 10^−3^	1.07 × 10^4^	−4.15 ± 1.18
0.81*	-	-	-
0.89*	-	-	-
1.00*	-	-	-

*^a)^F_PFS_* = molar composition of PFS in the poly(PFS-ran-MMA) copolymers dielectric layer. ^b)^ hole transport mobility (*µ*), the on-off current (*I_ON/OFF_*) and the threshold voltage (*V_T_*). *At these molar compositions, the CuPc no longer formed uniform films leading to device failure and irreproducible results.

## Supplementary Materials

The following are available online at https://www.mdpi.com/2073-4360/12/6/1231/s1, Figure S1. ^1^H NMR and ^19^F NMR spectrum of the final copolymer PFS/MMA 80/20 with a-a-a-trifluorotoluene marker. Figure S2. DSC exothermic thermograms of PFS/MMA copolymers. Figure S3. Dielectric constant versus frequency from 10^2^–10^5^ Hz obtained by impedance spectroscopy on MIM capacitors utilizing PFS/MMA copolymers. Figure S4. PFS/MMA copolymer OTFTs CuPc Visibility at varying PFS content. Figure S5. Microscope images of CuPc deposited on PFS copolymers. Figure S6. X-ray diffraction pattern performed on CuPc films deposited on PFS/MMA underlying films of varying compositions.
