# Supplementary material for: Controlled Synthesis of Poly(pentafluorostyrene-ran-methyl methacrylate) Copolymers by Nitroxide Mediated Polymerization and Their Use as Dielectric Layers in Organic Thin-film Transistors
Source: Polymers (Basel). 2020 May 29;12(6):1231. doi: 10.3390/polym12061231 (PMC7361672; doi:10.3390/polym12061231)
Supplement: Supplementary file 1 [file polymers-12-01231-s001.pdf]

# Controlled synthesis of poly(pentafluorostyrene-ran-methyl methacrylate) copolymers by nitroxide mediated polymerization and their use as dielectric layers in organic thin-film transistors

Alexander J. Peltekoff, Mathieu N. Tousignant, Victoria E. Hillier, Owen A. Melville, Benoît H. Lessard*.

Department of Chemical & Biological Engineering, University of Ottawa, 161 Louis Pasteur, Ottawa, ON, Canada K1N 6N5

Corresponding author: benoit.lessard@uottawa.ca

# SUPPORTING INFORMATION

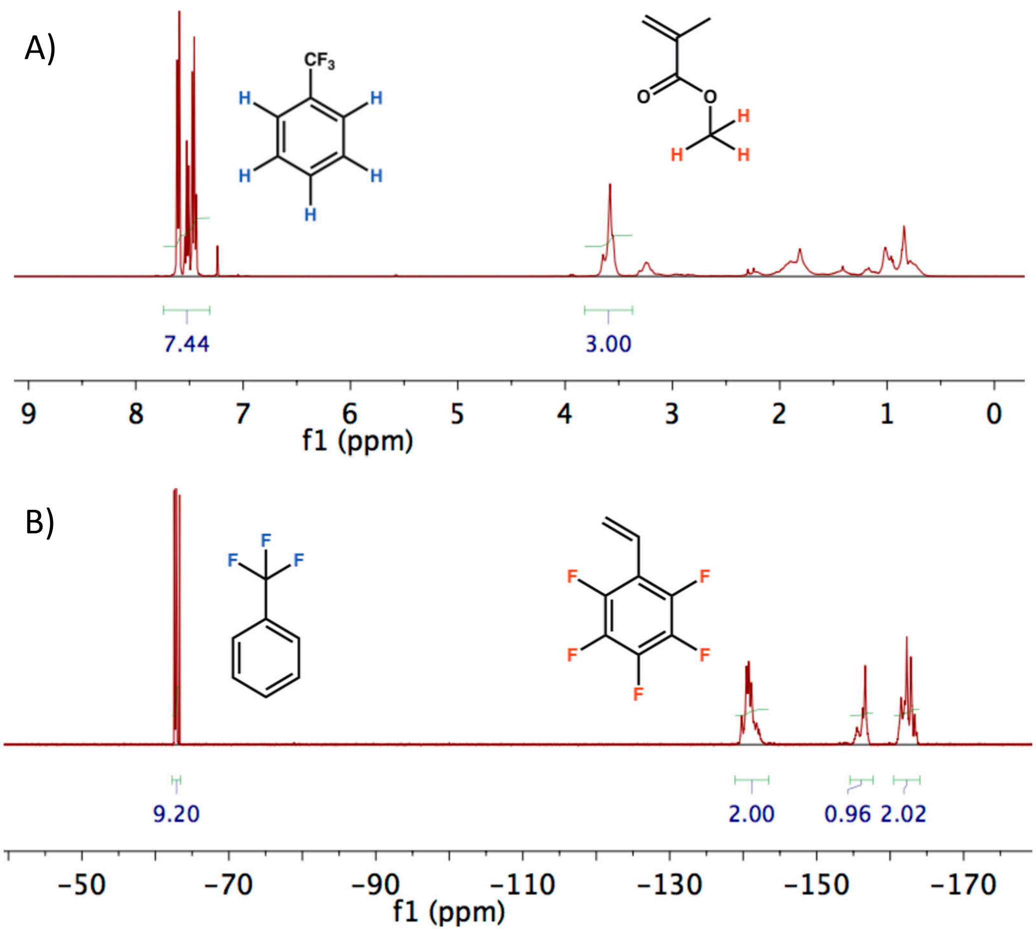


**Figure S1.** A) $^{1}$H NMR and B) $^{19}$F NMR spectrum of the final copolymer PFS/MMA 80/20 with α-α-α-trifluorotoluene marker MMA to TFT ratio of 7.44 : 3.00 and PFS to TFT ratio of 9.20 : 2.0.

The following is a derivation of the equation used to determine the molar composition of the copolymers using the same NMR solution and using TFT as a standard for NMR comparison. (Figure S1).

**$^{19}$F NMR:**

$$TFT = \frac{1}{3}\delta_{-63} \quad ; \quad PFS = \frac{1}{2}\delta_{-140} \quad ; \quad \frac{PFS}{TFT} = \frac{\left(\frac{1}{2}\delta_{-140}\right)}{\left(\frac{1}{3}\delta_{-63}\right)}$$

**$^{1}$H NMR:**

$$MMA = \frac{1}{3}\delta_{3.6} \quad ; \quad TFT = \frac{1}{5}\delta_{7.5} \quad ; \quad \frac{TFT}{MMA} = \frac{\left(\frac{1}{5}\delta_{7.5}\right)}{\left(\frac{1}{3}\delta_{3.6}\right)}$$

$$\frac{PFS}{MMA} = \frac{PFS}{TFT} \times \frac{TFT}{MMA} = \frac{\left(\frac{1}{2}\delta_{-140}\right)\left(\frac{1}{5}\delta_{7.5}\right)}{\left(\frac{1}{3}\delta_{-63}\right)\left(\frac{1}{3}\delta_{3.6}\right)}$$

$$F_{PFS} = \frac{PFS}{PFS\ +\ MMA} = \frac{\frac{PFS}{MMA}}{\frac{PFS}{MMA} + \frac{MMA}{MMA}} = \frac{\frac{\left(\frac{1}{2}\delta_{-140}\right)\left(\frac{1}{5}\delta_{7.5}\right)}{\left(\frac{1}{3}\delta_{-63}\right)\left(\frac{1}{3}\delta_{3.6}\right)}}{\frac{\left(\frac{1}{2}\delta_{-140}\right)\left(\frac{1}{5}\delta_{7.5}\right)}{\left(\frac{1}{3}\delta_{-63}\right)\left(\frac{1}{3}\delta_{3.6}\right)} + 1}$$

$$\boldsymbol{F_{PFS}} = \frac{\left(\frac{1}{2}\boldsymbol{\delta}_{-140}\right)\left(\frac{1}{5}\boldsymbol{\delta}_{7.5}\right)}{\left(\frac{1}{2}\boldsymbol{\delta}_{-140}\right)\left(\frac{1}{5}\boldsymbol{\delta}_{7.5}\right) + \left(\frac{1}{3}\boldsymbol{\delta}_{-63}\right)\left(\frac{1}{3}\boldsymbol{\delta}_{3.6}\right)}$$

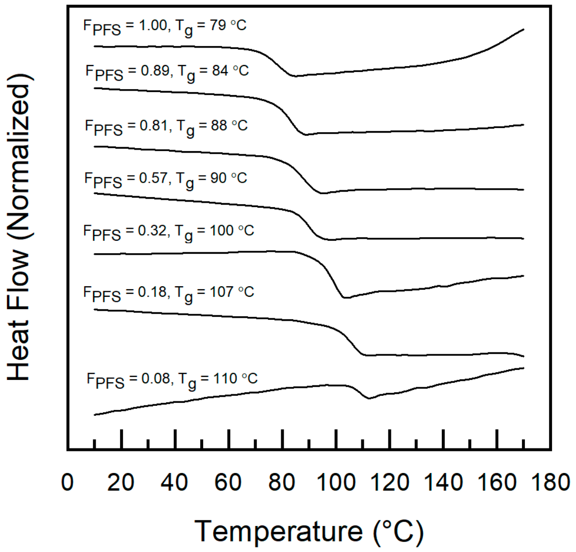


**Figure S2.** DSC exothermic thermograms of PFS/MMA copolymers

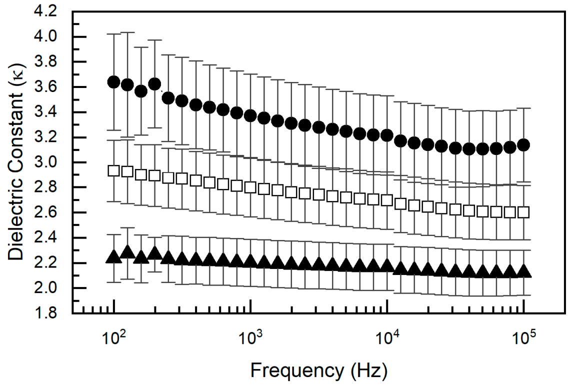


**Figure S3.** a) Dielectric constant versus frequency from $10^2 - 10^5$ Hz obtained by impedance spectroscopy on MIM capacitors utilizing PFS/MMA copolymers as insulating layer of compositions: $F_{PFS}$ = 0.08 (closed circles), $F_{PFS}$ = 0.57 (open squares), and $F_{PFS}$ = 0.81 (closed triangles).

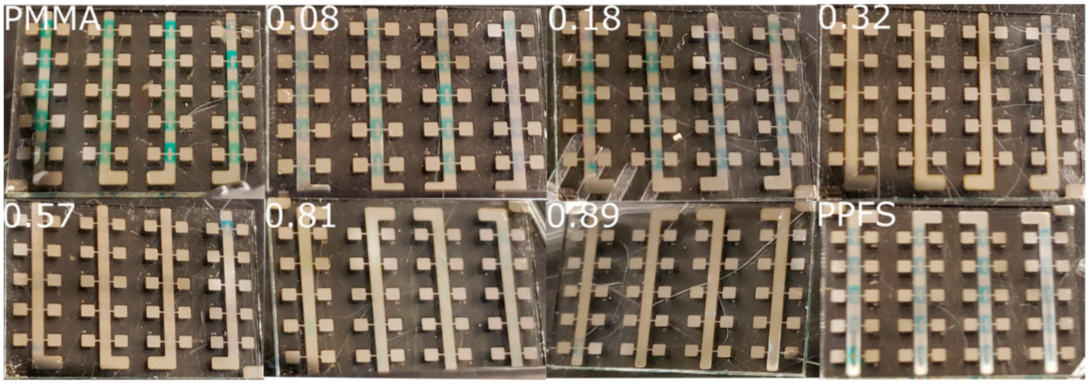


**Figure S4.** PFS/MMA copolymer OTFTs CuPc Visibility at varying PFS content.

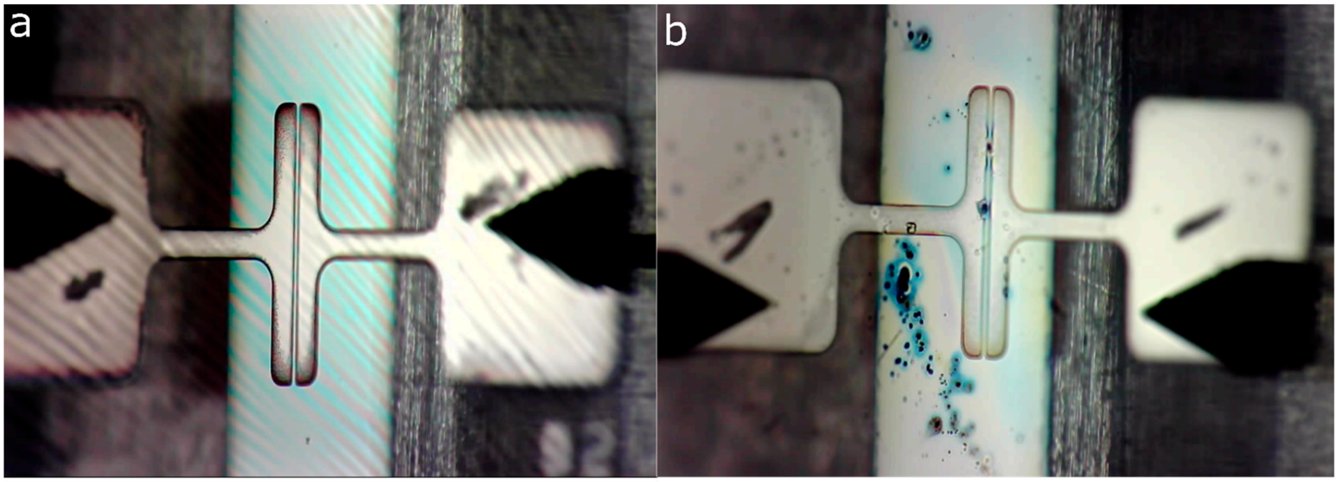


**Figure S5.** Microscope images of CuPc deposited on a). PFS 8 and b). PFS 81.

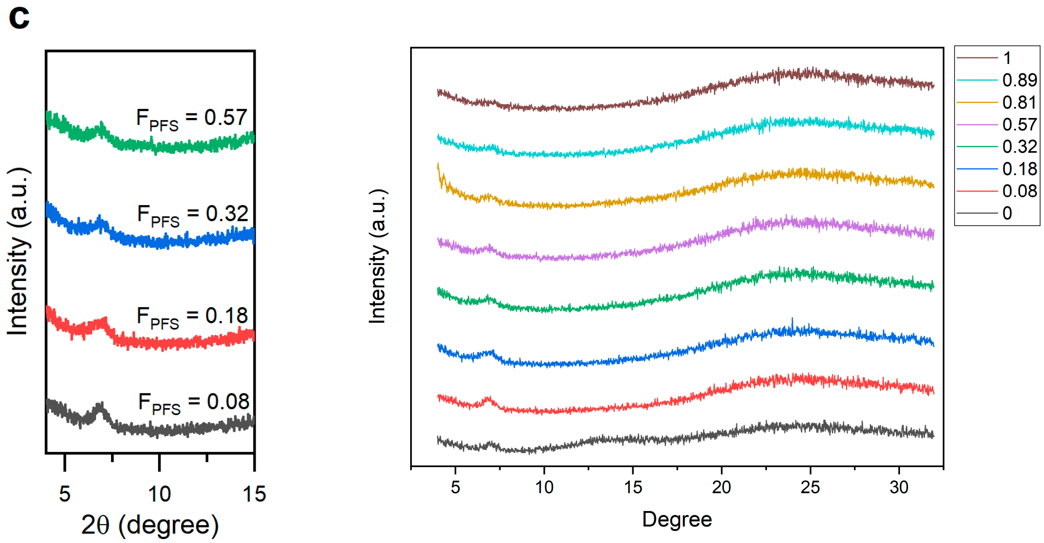


**Figure S6.** X-ray diffraction pattern performed on CuPc films deposited on PFS/MMA underlying films of varying compositions.
